# Psychometric properties of the Chinese version of the Amblyopia and Strabismus Questionnaire (ASQE)

**DOI:** 10.1186/s12955-015-0269-6

**Published:** 2015-06-12

**Authors:** Wei Bian, Min Li, Zonghua Wang, Xiaolei Wang, Yang Liu, Yan Wu

**Affiliations:** Southwest Hospital/Southwest Eye Hospital, Third Military Medical University, Chongqing, 400038 China; Key Lab of visual Damage and Regeneration & Restoration of Chongqing, Chongqing, 400038 China; School of Psychology, Third Military Medical University, Chongqing, 400038 China; School of Nursing, Third Military Medical University, Chongqing, 400038 China

**Keywords:** The Strabismus and Amblyopia Questionnaire, Quality of life, Chinese version, Psychometric properties

## Abstract

**Background:**

Strabismus and amblyopia are known to cause visual dysfunction, self-image disorders, difficulty in seeking employment and social and emotional barriers. These factors can have a serious and detrimental effect upon the patients’ health-related quality of life (HRQOL). Presently, a condition-specific questionnaire is not available for assessing the HRQOL in Chinese patients. This study developed a Chinese version of the Amblyopia and Strabismus Questionnaire (ASQE) and tested its reliability and validity in Chinese adult strabismus patients.

**Methods:**

Chinese strabismus adults, adults with normal vision and patients with a variety of other eye diseases completed the Chinese version of the ASQE. Reliability was established by Cronbach’s alpha and test-retest. Validity was evaluated by content, construct, criterion-related, convergent and discriminative validities.

**Results:**

A total of 202 adult strabismus patients with or without amblyopia, 100 visually normal adults, and 100 patients with other eye diseases (excluding strabismus and amblyopia) participated in this study. Using principal components analysis, six domains were extracted, with a content validity of 0.91. Four items were deleted giving final total of 22 items in the questionnaire. The total score of the ASQE was significantly correlated to the Adult Strabismus Questionnaire (AS-20) (r = 0.642, *P* < 0.01). The median scores for the adult strabismus patients were significantly lower (worse HRQOL) compared with visually normal adults (66.32 vs. 92.71; *P* < 0.001) and patients with other eye diseases (66.32 vs. 79.50; *P* < 0.001) thus demonstrating good discriminative validity for the questionnaire. The Cronbach’s alpha coefficient for internal consistent reliability was 0.887 and the test-retest reliability was 0.946. The mean total score of the ASQE was 65.85 (SD = 15.32) and the domain ‘social contact and appearance’ recorded the lowest mean score 43.78 (SD = 13.92) in strabismus patients.

**Conclusions:**

The revised 22-item Chinese version of the ASQE showed good psychometric properties. It is suggested that this questionnaire provides a potentially useful measurement tool in clinical or research programs involving Chinese strabismus patients with or without associated amblyopia.

## Background

Strabismus is a common disease in which the eyes are inproperly aligned, pointing in different directions. Patients can easily adapt to this condition by suppressing images from one of their eyes, eliminating the sensation of double vision. However, this plastic response of the brain, interrupts normal development and results in the potential development of amblyopia. In contrast to strabismus, the non-dominant eye in amblyopia becomes essentially non-functional. Strabismus and amblyopia are known to cause visual dysfunction, self-image disorders, difficulty in seeking employment and social and emotional barriers. These factors can have a serious and detrimental effect upon the patients’ health-related quality of life (HRQOL) [1–5]. Additionally, children as young as five or six years of age may react negatively to peers with obvious symptoms strabismus [6–8]. Even in adult strabismus patients whose eyes remain successfully aligned, with improved levels of both psychosocial and functional scores from 6 weeks to 1 year postoperatively, problems such as visual confusion and reading remain [9]. This is especially true in strabismus patients with diplopia, where no improvement in function and psychosocial scores has been noted one year postoperatively [9]. Recently, a large number of HRQOL instruments have been developed and applied in research and clinical ophthalmic settings. These include the 25-Item National Eye Institute Visual Function Questionnaire (NEI-VFQ-25) [10], the Low Vision Quality-of-life Questionnaire (LVQOL) [11], the Activities of Daily Vision Scale (ADVS) [12], and the Amblyopia Treatment Index Parental Questionnaire (ATI) [13].

Unlike generic instruments, the Amblyopia and Strabismus Questionnaire (ASQE) was the first patient-derived and condition-specific questionnaire for strabismus and/or amblyopia to be shown to be a valid and reliable instrument in several studies [14–17]. The original Dutch-language version [16], based on the experience of amblyopia and strabismus patients in the Netherlands, demonstrated high internal consistency with a Cronbach’s alpha ranging from 0.76 to 0.93 for each domain, discriminating between healthy controls from the Waterland patient study group. Van de Graaf *et al.* [17] used the ASQE to evaluate the quality of life in patients who were treated by occlusion therapy, showing that visual acuity was significantly correlated with all the domains in a cohort of 174 patients. In addition, the high level of variance in ASQE scores by the six factors found by the PCA confirmed the a priori hypothesized dimensions of this HRQOL instrument [15]. The English language version of the ASQE showed good psychometric properties and had a high correlation with the domains of the disability questionnaire including; the impact of the visual problems on specific health issues, daily functioning, social interaction, concerns about the future, self-image and job-related difficulties [14]. Bujak *et al.* [18] used the ASQE to test the quality of life for strabismus patients and found their scores and daily function improved after monoocular correction, demonstrating good discriminative validity. In summary, the ASQE not only potentially captures the impact of visual and appearance problems on physical, psychological and social functioning, but also provides an evidence-based basis for developing personalized medical and nursing care for these patients.

To date the HRQoL of Chinese strabismus and amblyopia patients remains poorly understood, mainly as there is no disease-specific instrument for them. Although the ASQE demonstrates excellent psychometric properties, the questionnaire has not been translated and validated in Chinese, which is one of the most widely used languages in the world. This study developed a Chinese version of the ASQE and tested its reliability and validity in Chinese adult strabismus patients.

## Methods

### Participants and study setting

This study employed a cross-sectional, descriptive design. A convenient and consecutive sample was obtained from the outpatient clinic and ward of the Southwest Eye Hospital and consisted of a total of 202 adult strabismus patients with or without amblyopia, 100 normal adults, and 100 patients with other eye diseases (excluding strabismus and amblyopia). The inclusion criteria for the strabismus patients were as follows: (1) age ≥ 18 years with obvious strabismus (with or without amblyopia) for more than 3 months; (2) patients did not have any other facial or ocular abnormalities, or acute eye diseases; (3) patients were not taking any anti-anxiety or antidepressant medication; (4) patients had good visual acuity (20/50 or better) in their better-seeing eye. Patients with poor visual acuity in both eyes were excluded to prevent large quality of life influences resulting from losing sight; (5) patients with a previous history of any surgical intervention were also excluded to avoid data bias.

The normal sample consisted of 64 family members or companions of the strabismus patients and 36 university students recruited into the study. Also, 100 patients with other eye diseases participated into the study including retinal detachment (n = 26), vitreous hemorrhage (n = 19), cataract (n = 24), glaucoma (n = 14) and ocular trauma (n = 17). All of the normal people and patients with other eye diseases had no history of strabismus or amblyopia. There were no statistically significant demographic differences between the three study groups according to the distribution of age, gender, marital status, or education.

The visual acuity and a diagnosis of strabismus were assessed and provided by a ophthalmic physician for each patient. They were classified as diplopia, non-diplopia, amblyopia, or non-amblyopia based on their past medical history and clinical records.

### Instruments

#### Amblyopia and Strabismus Questionnaire (ASQE)

The original ASQE contains a total of 26 items divided into six domains. These are: (1) fear of losing the better eye, (2) far distance estimation, (3) near distance estimation, (4) visual disorientation, (5) double vision, and (6) social contact and appearance. The responses for each item were measured on a Likert-type rating scale ranging from never (score 100), rarely (score 75), sometimes (score 50), often (score 25), and always (score 0). The total score is the mean of all the questions answered. The highest score is 100 indicating the best possible HRQOL, while the lowest is 0 indicating the worst possible HRQOL.

#### Adult Strabismus Questionnaire (AS-20)

The AS-20 is a strabismus-specific questionnaire with a total of 20 items divided into two domains. The first domain is ‘psychosocial’ which measures the psychosocial functioning and self-awareness. The second domain is ‘function’, covering physical and emotional functions. Each domain consists of 10 items rated on the 5-point Likert-type scale for all the responses: never (score 100), rarely (score 75), sometimes (score 50), often (score 25), and always (score 0), respectively. The overall score is the mean of all the questions answered. For the Chinese version of the AS-20, the Cronbach’s alpha was 0.908 for the overall scale, with 0.913 and 0.808 for the ‘psychosocial’ and ‘function’ domains, respectively [19].

### Translation and Development of the Questionnaire

The original ASQE is available free of charge at http://www.Retinafoundation.org/questionnaire.html. Permission was obtained from the ASQE copyright holder, Professor Simonsz, followed by a standard forward-backward translation procedure conducted to develop the Chinese version of the ASQE [20]. Initial translation, from English to Chinese, was undertaken by an ophthalmology professor and a professional translator without medical or clinical background. These translations were then compared to avoid discrepancies or any ambiguous wording from the translation process. Discussion was held to resolve any inappropriate choice of words. Different profiles were completed including the instructions, item content and options and rationale for their choices to solve any uncertainties or challenging phrases.

The two translated versions were synthesized by the two translators and a recording observer to reach a consensus on a single Chinese version. In addition, a written report was prepared to document the process and highlight how issues were overcome. The back-translation was conducted by professional translators, who were unfamiliar with the questionnaire and blind to the original version. All versions of forward and back translated ASQE were compared and consolidated to develop the prefinal version of the questionnaire. The process was conducted rigorously to ensure idiomatic, semantic, experiential and conceptual equivalence, including adaptation of the language to respect cultural considerations.

Fifty adult strabismus patients were recruited for a pilot test to ensure the equivalence of the scale in an applied situation. Interviews were made to find what the patients thought about the items and options. Continued revisions were made until an finally acceptable translated Chinese version was agreed upon.

### Data collection

Patients meeting the inclusion criteria were recruited on their first day of hospitalization or while visiting the outpatient clinics prior to surgery. All participants were given written and verbal instructions by the researcher, including an overview of the study content, objectives and overall purpose of the study. The adult strabismus patients completed a history data sheet (detailing the visual acuity of the eyes, the type of strabismus and whether this was with or without double vision or amblopia, and the patient’s age, gender, and marital status), the ASQE and AS-20 questionnaires. The normal adults and other eye disease patients also completed the ASQE questionnaire. To assess the test-retest reliability, 50 strabismus patients were randomly chosen and were asked to complete the ASQE again seven days later; all tests were done prior to subsequent strabismus surgical intervention or treatment. All the questionnaires were self-administered.

### Data analysis

#### Reliability and validity

Data were analyzed by the SPSS statistical package (SPSS, Version 20.0; significance *P* ≤ 0.05 two tailed test) and checked by two researchers to ensure its accuracy. The Kolmogorov-Smirnov test was used to examine the normal distribution of the data and descriptive statistics were used to summarize patient characteristics.

Extreme group and correlation analysis methods were used as the criterion for judgment in item analysis. Subjects were categorized into two groups according to their scores and then the differences between the scores compared with a rank-sum test; the highest 27 % composed the high scoring group, while the lowest 27 % composed the low scoring group. Analyses were assessed by differences between the scores of all items of the two extreme groups. Correlation between each item and the total score were evaluated using Spearman’s rank correlation.

A content validity index (CVI) [21] was used to determine item validity. A panel of experts was asked to rate each item of the Chinese version of the ASQE on a 4-point scale of 1 (not relevant), 2 (somewhat relevant), 3 (relevant), or 4 (very relevant). The CVI was calculated using the percentage of items receiving a rating of 3 or 4, and a CVI value exceeding 0.80 indicated good content validity [22].

Construct validity was statistically assessed by means of principal component factor analysis with varimax rotation, the Kaiser-Meyer-Olkin Measure of Sampling Adequacy (KMO), and calculation of the Bartlett’s Test of Sphericity.

Criterion–related validity was performed by calculating the correlation between the ASQE and AS-20 questionnaires.

Convergent validity was evaluated by calculating correlations between the scores of each domain and the total score.

Discriminative validity was evaluated through comparison of the median scores of the adult strabismus patients with those of the normal adults or patients with other eye diseases using the Mann–Whitney *U* test.

For reliability, internal consistency was estimated by the Cronbach’s alpha coefficient and test-retest reliability was assessed with the intra-class correlation coefficient. A Cronbach’s alpha ≥ 0.70 was considered adequate [22].

The differences in quality of life among strabismus patients with or without amblyopia were tested, according to age, gender and clinical features, using one-way ANOVA analyses or independent-samples t tests.

## Results

### Participant characteristics

The demographic and clinical characteristics of the strabismus patients, visually normal adults and patients with other eye disease are summarized in Table 1.

**Table 1:** Demographic and clinical Characteristics of the strabismus patients, visually normal adults and patients with other eye diseases

Group	Strabismus	Normal vision	With other eye disease
	(n = 198)	(n = 100)	(n = 100)
Age, years (range)	25 (18 ~ 67)	26(18 ~ 57)	29(18 ~ 69)
Sex, n			
Male	98	47	45
Female	100	53	55
Strabismus type, n (†PACT)			
Esotropia	90(30PD)	N/A	N/A
Exotropia	72(48PD)	N/A	N/A
Vertical	46(18PD)	N/A	N/A
Amblyopia, n			
With	94	0	0
Without	104	100	100
Visual Acuity			
Better eye	20/15-20/30	20/25-20/15	20/20-20/50
Worse eye	20/15-20/50	20/25-20/15	20/20-20/80

† mean Prism and alternating cover test (PACT) values in prism diopters (PD) are shown in brackets

N/A, not applicable

A cohort of 202 strabismus patients (with or without amblyopia) participated in the study. All questionnaires were returned, but four were incorrectly completed, leaving a total sample of 198 subjects. Nearly half of the strabismus patients (n = 98) were male, 22.7 % suffered from diplopia (n = 45) and 24.8 % had amblyopia (n = 94). Visual acuity ranged from 20/15 to 20/30 (median, 20/20) for the better eye and 20/15to 20/50 (median, 20/30) for the other eye. The median angle of deviation measured at distance by a prism and an alternating cover test (PACT) was 30 prism diopters (PD; range, 15 ~ 80) for the 90 patients with primary esodeviation. Patients with primary exodeviation (n = 72), had a median PACT of 48 PD (range 15 ~ 74), and 46 patients with vertical deviation had a median PACT of 18 PD (range 8 ~ 45).

All visually normal patients and those with other eye diseases returned valid questionnaires, they had no more than 10 PD of horizontal and 1 PD of vertical heterophoria. In the normal subjects, stereoacuity was 40–80 sec of arc using the TNO test, and their best-corrected visual acuity was at least 20/25 (median 20/20 for each eye). For subjects with other eye diseases, visual acuity ranged from 20/20 to 20/50 (median, 20/30) for the better eye and 20/20 to 20/80 (median, 20/40) for the other eye.

### Judgment in items analysis

The extreme group analysis showed significant differences between the scores of all items of the two extreme groups (*P* < 0.001).

The coefficient correlation between each item to the total score ranged from 0.347 to 0.672 (*P* < 0.001) except for item 24, i.e. ‘My eyes are misaligned (one or both eyes cross, or turn out or turn up)’ , which had a correlation coefficient under 0.2, as such this item was deleted from further analysis.

### Content validity

The average CVI was 0.91 for the total scale, indicating an adequate level of content validity. The experts agreed that the ASQE questionnaire was conceptually and culturally relevant to measure the physical, psychological and social changes encountered by strabismus patients. However, with specific regard to items 14 to 16 the option to choose ‘Not relevant…’ was deleted due to local conditions and habits and to make the scale scoring more reasonable, or due to a lack specific relevance to the patients e.g. ‘Not relevant, because I do not have misaligned eyes’ as in items 25 and 26, given that all these study patients have strabismus.

### Construct validity

The Bartlett’s test for sphericity was significant (*P* < 0.001) and the Kaiser-Meyer-Olkin measure of sampling adequacy was 0.854, which indicated that it was appropriate to use the principal component analysis for all the data [23]. Six factors with eigenvalues greater than 1.0 were extracted, explaining 67.6 % of the total variance. When the total variance was examined, the 1^st^ factor, ‘far distance estimation’ accounted for 20.0 % of the total variance, the 2^nd^ factor, ‘social contact and appearance’ accounted for 10.5 %, the 3^rd^ factor, ‘visual disorientation’ accounted for 10.1 %, the 4^th^ factor, ‘near distance estimation’ accounted for 9.2 %, the 5^th^ factor, ‘double vision’ accounted for 9.1%, and the 6^th^ factor, ‘fear of losing the better eye’ accounted for 8.7 %. All items except for items 13, 19 and 20 demonstrated moderate to strong loading on one of the six factors (>0.4; See Table 2).

**Table 2:** Factor loading, eigenvalues, and percent of variance for ASQE items emerging from the principal components analysis

Item	Description	Factors					
		1	2	3	4	5	6
6	I feel unsure or hesitant when putting something on a table	0.738					
7	I miss the other person’s hand when trying to shake hands.	0.811					
8	I have difficulty parking my car.	0.712					
9	I find it difficult to put the cap on a pen or marker.	0.795					
10	I find it difficult to put a power plug into a socket.	0.828					
11	I have difficulties pouring drinks.	0.809					
12	I have difficulties walking down stairs.	0.589				0.306	
22	I have difficulty making eye contact in a one-on-one conversation.		0.878				
23	I have difficulty making eye contact with people in a group conversation.		0.907				
25	Because of my misaligned eyes I feel insecure.		0.585				
26	If I did not have misaligned eyes, I would have more self-confidence.		0.574				0.327
14	I have difficulties finding my way in a shopping mall, especially when I am there for the first time.			0.839			
15	I have difficulties finding my way in a department store or a supermarket, especially when I am there for the first time.	0.301		0.807			
16	I have difficulties finding my way in a train station, especially when I am there for the first time.			0.774		0.301	
4	I can estimate distances well.				0.775		
5	I have good depth perception				0.735		
21	I have to squint or shut one eye in bright sunlight.		0.344		0.590		
13	I have difficulties playing ball games.	0304		0.316	0.364		
17	I see double.					0.891	
18	Double vision disturbs me in my daily activities (household, study, school, hobbies, work).	0.304				0.859	
19	When I am tired, I must be very careful not to miss what I reach for.	0.395		0.340		0.421	
20	I have to do things more slowly when I am tired because of my eyesight.	0.371			0.430	0.441	
1	I can see equally well with both eyes.						0.683
2	I worry about losing my better eye.						0.843
3	I worry that something might get into my better eye.						0.814
Eigenvalues		4.996	2.614	2.529	2.309	2.275	2.173
Variance explained (%)		19.983	10.458	10.118	9.236	9.100	8.693
Cumulative variance explained (%)		19.983	30.441	40.559	49.795	58.895	67.588

Only factor loading ≥0.3 are shown

### Criterion-related validity

The criterion-related validity showed a strong positive correlation (r = 0.582, *P* < 0.01) between the ASQE and AS-20.

### Convergent validity

Correlation coefficients were calculated to compare the scores of each domain and the whole score. Table 3 shows that the ASQE had a low and moderate correlations between the scores of each scale with r ranging from 0.167 to 0.513 (*P* < 0.05), but had a high correlation between the score of each domain with the total index score, which ranged from r = 0.566 to 0.658 (*P* < 0.01) indicating adequate convergent validity.

**Table 3:** Correlations between scores for each scale and the total score

	Description	1	2	3	4	5	6
1	Far distance estimation	1					
2	social contact and appearance	0.260**	1				
3	visual disorientation	0.434**	0.270**	1			
4	near distance estimation	0.513**	0.269**	0.319**	1		
5	double vision	0.372**	0.167**	0.366**	0.329**	1	
6	fear of losing the better eye	0.286**	0.232**	0.272**	0.238**	0.257**	1
7	Total score	0.598**	0.566**	0.652**	0.612**	0.658**	0.653**

Spearman coefficient test. ** *P* <0.01

### Discriminative validity

Discriminative validity was used to evaluate how well the questionnaire discriminated between strabismus patients, visually normal adults, and patients with other eye diseases. The median scores for the adult strabismus patients were significantly lower (worse HRQOL) compared with visually normal adults (66.32 vs. 92.71; *P* < 0.001) and patients with other eye diseases (66.32 vs. 79.50; *P* < 0.001). The median score was also significantly lower for patients with other eye diseases when compared with visually normal adults (79.50 vs. 92.71; *P* < 0.001).

### Reliability

Cronbach’s alpha coefficient was used to define internal consistency and showed that the refined 22-item Chinese ASQE had a coefficient of 0.887, indicating good internal consistency or homogeneity; the values for all the domains ranged from 0.663 to 0.920. The correlation coefficient analysis examining the test-retest reliability for the subset of strabismus patients tested after a one-week interval was 0.946 for the overall score and 0.730 ~ 0.953 for the domain (*P* < 0.01; Table 4).

**Table 4:** Reliability of the ASQE (n = 198)

Scale (items)	Test-retest reliability	Internal consistency reliability
	(n = 50)	(n = 198)
far distance estimation(7)	0.730	0.908
social contact and appearance(4)	0.905	0.771
visual disorientation(3)	0.811	0.663
near distance estimation(3)	0.834	0.848
double vision(2)	0.916	0.920
fear of losing the better eye(3)	0.953	0.777
Overall scale(22)	0.946	0.887

### Patients’ demographic factors on ASQE

There were no significant differences in the total scores between the sexes, or patients with or without diplopia and/or amblyopia (see Table 5). The male participants had higher scores on the ‘social contact and appearance’ domain (*P* = 0.001) than females. Strabismus patients with amblyopia had lower scores on the ‘far distance estimation’, ‘social contact and appearance’ and ‘visual disorientation’ domains (*P* < 0.05) compared to those patients without amblyopia. In addition, patients with diplopia reported lower scores on the ‘far distance estimation’ and ‘visual disorientation’ domains (*P* < 0.05), compared with patients without diplopia. No significant correlation was found between the scores and age or visual acuity in the better-seeing eye (two-tailed Spearman correlation coefficient −0.069; *p* = 0.33).

**Table 5:** Comparison of mean ASQE according to sex and in strabismus with or without diplopia and amblyopia (n = 198)

	ASQE mean score (SD)						
	Total	Factor 1	Factor 2	Factor 3	Factor 4	Factor 5	Factor 6
Gender							
Male	67.11(15.64)	90.16(15.76)	49.62(13.61)**	82.14(18.50)	55.78(13.25)	78.70(18.22)	46.26(18.45)
Female	64.02(14.97)	89.75(15.60)	38.06(12.93)	80.42(10.93)	52.25(12.96)	77.25(19.96)	50.00(12.11)
Amblyopia							
With	64.30(15.06)	84.90(16.00)**	39.30(12.29)*	74.61(10.69)*	52.66(10.96)	76.73(19.42)	48.67(10.71)
Without	66.24(15.49)	94.90(15.32)	47.84(14.71)	87.77(18.81)	55.21(14.94)	79.09(18.80)	47.68(10.14)
Diplopia							
With	67.09(14.30)	84.31(16.02) *	42.22(16.12)	72.51(10.91)*	53.89(10.99)	85.56(12.45)	52.04(10.68)
Without	65.49(15.63)	95.85(15.60)	44.24(13.30)	88.08(19.37)	54.03(13.77)	75.74(10.42)	47.00(10.24)
Total	65.85 (15.32)	89.95 (15.64)	43.78 (13.92)	81.27(19.74)	54.00(13.11)	77.97(19.05)	48.15(10.33)

Factor 1: far distance estimation; Factor 2: social contact and appearance; Factor 3: visual disorientation; Factor 4: near distance estimation; Factor 5: double vision; Factor 6: fear of losing the better eye

** *P* <0.01; * *P* <0.05

### The ASQE score on the total and domains

The mean total score for the ASQE was 65.85 (SD = 15.32). The domains, showing ‘social contact and appearance’ had the lowest mean score 43.78 (SD = 13.92), while the ‘far distance estimation’ showed the highest mean score 89.95 (SD = 15.64) (See Table 5).

## Discussion

The Chinese version of the ASQE contains 22 items in six domains and was developed specifically for adult Chinese strabismus patients with or without amblyopia. The data presented in our study indicates that the ASQE has good psychometric properties and is culturally appropriate for evaluating the HRQOL among strabismus adults in China.

With regards to reliability, indicate that the Chinese version of the ASQE has a high internal consistency with a Cronbach’s alpha of 0.887 for the overall score, and suggests high internal homogeneity. The test-retest value was 0.946 for the overall score and 0.730 ~ 0.953 for the domains, indicating its high stability over time. In addition, the total correlation coefficients for all 22 items ranged from 0.347 to 0.672, which meant the items were considered discriminative from the total index and correlated with the total scale. All of the above findings would suggest that ASQE is a reliable and stable instrument for evaluating the HRQOL among strabismus and amblyopia patients in China.

For the construct validity, our findings suggest that items 13, 19, 20 and 24 (see Table 2) could be deleted to keep model fitness, and as such these items were excluded from further examination. Six factors were extracted, which included ‘far distance estimation’, ‘social contact and appearance’, ‘visual disorientation’, ‘near distance estimation’, ‘double vision’, and ‘fear of losing the better eye’. This finding is consistent with that of van de Graaf and colleagues [15], although some items were classified into dimensions differently. For example, in the original ASQE, the item ‘I have difficulty parking my car’ belonged to the factor ‘near distance estimation’, but was moved to ‘far distance estimation’ in our study due to potential cultural differences. However, six factors were identified in our study as opposed to seven factors previously reported in an Italian version which had separated the ‘social contact and appearance’ into ‘social contact’ and ‘appearance’ parts. This difference may be potentially explained by the use of different samples, cultural and social difference between Italian and Chinese populations [24]. Due to a lack of data from previous research on ASQE factor analysis, patients from China and other countries can not be directly compared. It is suggested that future studies aim to verify and confirm the different factor model.

Criterion–related validity was performed by calculating the correlation between the ASQE and AS-20 questionnaires. Recently, the Chinese version of the Adult Strabismus Quality of Life Questionnaire (AS-20), has been developed and is now widely used in testing the functional status and factors that influence quality of life in strabismus patients preoperatively and postoperatively [19, 25, 26]. We have chosen the AS-20 for our Criterion-related validity because these two original questionnaires are patient-derived, both are measured on a Likert-type rating scale and they are of similar design and format. Additionally, there are no strabismus specific instruments that can currently be considered as a ‘gold standard’ measurement in China, and the AS-20 is better than any other questionnaire available as a Chinese version. The results showed the correlation coefficient between the ASQE and AS-20 was 0.582 indicating good Criterion-related validity. Further studies are needed to compare the Chinese version of the two questionnaires to fully explore their role in clinical practice.

We analyzed the median overall scores for strabismus patients, normal adults and patients with other eye diseases to test discriminative validity and found a significant difference between groups. The results demonstrated that strabismus patients had a lower median HRQOL score compared with the two control groups, which was consistent with the results of van de Graaf *et al.* and Marcon *et al.* [16, 24]. Although the patients with other eye diseases had significantly lower overall scores compared with normal adults, the magnitude of the difference was much smaller when compared to that between strabismus patients and normal adults. This indicates that the ASQE contains several HRQOL-related items that are not only concerned with visual acuity, but also includes items related to self-image and appearance disorders, which have an impact on the Chinese person’s reputation and recognition [27] and may not be relevant or appear among patients with other eye disease patients.

Clinical features and demographic factors were also taken into consideration whilst testing the validity of questionnaire. We found that females scored lower on the ‘social contact and appearance’ scale than the males, and hypothesize that this is may be partly due to idealized images of beauty and greater sensitivity to societal reactions [28]. Females have greater concerns about appearance and related self-esteem issues, both of which are very important in Chinese culture. Strabismus and the appearance of misaligned eyes may induce ‘loss of face’ and feelings of inferiority. This clearly shows the impact of societal attitudes on the HRQOL for these patients. Additionally, strabismus patients with amblyopia had lower scores on the ‘far distance estimation’, ‘social contact and appearance’ and ‘visual disorientation’ domains compared with patients without amblyopia, indicating that patients with amblyopia not only suffer from functional problems but also had a greater level of psychosocial concern; a factor possibly overlooked by healthcare clinicians. We also found that patients with diplopia had lower scores on the ‘far distance estimation’ and ‘visual disorientation’ domains compared with patients without diplopia, but there were no significant differences on the ‘social contact and appearance’ scales. There was no significant correlation between scores and age, partly because most of our strabismus patients are very young (median age: 25 years) who are perceived to be the most important person in a ‘one child’ Chinese family unit. Since the strabismus will bring them many problems such as communication barrier, seeking job and destroying their reputation in the society, they tend to visit the doctor and have the surgery at an early age. Future studies should include a larger cohort of older patients and compare the differences of the two groups. Also, there were no significant correlation between scores and visual acuity, this is most likely due to the good visual acuity of the better eye in all participants.

The mean ASQE score of 65.85 was consistent with the findings of van de Graaf *et al.* [16], indicating that adult strabismus and amblyopia patients commonly experienced physical and psychosocial problems. Among all the domains, ‘social contact and appearance’ rated the lowest score in the Chinese ASQE, which demonstrated that patient outlook and social functioning were rated as their most serious concerns. This may be due to encountering many social difficulties as a result of a lack of eye contact and nonverbal communication [29–31]. However, this view differs slightly from the findings of van de Graaf and coworkers [16] in which double vision was the biggest problem in the Ophthalmology Outpatient Clinic of Erasmus Medical Center, Rotterdam, Netherland. This difference may be explained on the basis that the Chinese advocate a high degree of social evaluation and of the requirement to make eye contact, while in other studies there may be a bias towards self-awareness and personal freedom, showing that their main concern may be with the condition itself, rather than the views of others.

A potential limitation of this study was a patient selection bias. Strabismus patients came to the hospital for surgery because of dissatisfaction or even self-abasement with regard to their appearance and image, thus, our results may not be representative of the wider strabismus and amblyopia cohort in China, some of whom may not have such concerns. Additionally, this study used classical test theory to assess the psychometric properties of the ASQE. Modern psychometric theory, such as Rasch analysis and confirmatory factor analysis were not used. We will use Rasch analysis and confirmatory factor analysis with a larger sample size to ensure the robust, scientific measurement of the ASQE and compare the outcomes in future research. Long-term studies would be preferable, however, it was difficult to test the HRQOL for extended periods of time after the surgery because of a high dropout rate. Further studies should be used to estimate its sensitivity to changes between pre- and postoperative HRQOL.

## Conclusions

In summary, the revised Chinese version of the ASQE appears a reliable and valid instrument providing valuable information for research on clinical therapy, medical care intervention, and health services in the Chinese context. Further studies are needed to find the explicit impact factors that influence the quality of life among strabismus and amblyopia patients, as well as assessing long term pre- and postoperative histories. This information will help clinicians and nursing staff to recommend appropriate treatment strategies, including surgical or psychiatrics modalities to maximize these patients overall HRQOL.

### Ethics consideration

Approval was obtained from the Ethics Committee of First Affiliated Hospital of Third Military Medical University. All procedures were performed after informing the patients and obtaining written consent, and were conducted according to the standards of the Declaration of Helsinki.

## Abbreviations

HRQoL: Health-related quality of life
(ASQE): Amblyopia and Strabismus Questionnaire
(AS-20): Adult Strabismus Questionnaire
CVI: Content validity index
